# In Vitro Metabolism of DWP16001, a Novel Sodium-Glucose Cotransporter 2 Inhibitor, in Human and Animal Hepatocytes

**DOI:** 10.3390/pharmaceutics12090865

**Published:** 2020-09-11

**Authors:** Ju-Hyun Kim, Dong Kyun Kim, Won-Gu Choi, Hye-Young Ji, Ji-Soo Choi, Im-Sook Song, Sangkyu Lee, Hye Suk Lee

**Affiliations:** 1 College of Pharmacy, Yeungnam University, Gyeongsan 38541, Korea; jhkim@yu.ac.kr; 2 BK21 PLUS Team for Creative Leader Program for Pharmacomics-Based Future Pharmacy, College of Pharmacy, The Catholic University of Korea, Bucheon 14662, Korea; kdk3124@catholic.ac.kr (D.K.K.); cwg0222@catholic.ac.kr (W.-G.C.); 3 Life Science Institute, Daewoong Pharmaceutical Co. Ltd., Yongin 17028, Korea; hychi138@daewoong.co.kr (H.-Y.J.); jschoi172@daewoong.co.kr (J.-S.C.); 4 College of Pharmacy, Research Institute of Pharmaceutical Sciences, Kyungpook National University, Daegu 41566, Korea; isssong@knu.ac.kr

**Keywords:** DWP16001, SGLT2 inhibitor, metabolism, CYP, UGT

## Abstract

DWP16001 is currently in a phase 2 clinical trial as a novel anti-diabetes drug for the treatment of type 2 diabetes by selective inhibition of sodium-glucose cotransporter 2. This in vitro study was performed to compare the metabolism of DWP16001 in human, dog, monkey, mouse, and rat hepatocytes, and the drug-metabolizing enzymes responsible for the metabolism of DWP16001 were characterized using recombinant human cytochrome 450 (CYP) and UDP-glucuronosyltransferase (UGT) enzymes expressed from cDNAs. The hepatic extraction ratio of DWP16001 in five species ranged from 0.15 to 0.56, suggesting that DWP16001 may be subject to species-dependent and weak-to-moderate hepatic metabolism. Five phase I metabolites (M1–M5) produced by oxidation as well as three DWP16001 glucuronides (U1–U3) and two hydroxy-DWP16001 (M1) glucuronides (U4, U5), were identified from hepatocytes incubated with DWP16001 by liquid chromatography-high resolution mass spectrometry. In human hepatocytes, M1, M2, M3, U1, and U2 were identified. Formation of M1 and M2 from DWP16001 was catalyzed by CYP3A4 and CYP2C19. M3 was produced by hydroxylation of M1, while M4 was produced by hydroxylation of M2; both hydroxylation reactions were catalyzed by CYP3A4. The formation of U1 was catalyzed by UGT2B7, but UGT1A4, UGT1A9, and UGT2B7 contributed to the formation of U2. In conclusion, DWP16001 is a substrate for CYP3A4, CYP2C19, UGT1A4, UGT1A9, and UGT2B7 enzymes. Overall, DWP16001 is weakly metabolized in human hepatocytes, but there is a potential for the pharmacokinetic modulation and drug–drug interactions, involved in the responsible metabolizing enzymes of DWP16001 in humans.

## 1. Introduction

In 2019, approximately 463 million adults (20–79 years of age) were living with diabetes [1]. Without appropriate control of blood glucose level, diabetes can lead to cardiovascular and microvascular complications. Therefore, various medications (e.g., insulin, glucagon-like peptide-1 analogues, metformin, sulfonylureas, thiazolidinediones, and dipeptidyl peptidase-4 inhibitors) have been used for the treatment of type 2 diabetes.

Sodium-glucose cotransporter 2 (SGLT2) is important in renal glucose reabsorption; selective blockade of SGLT2 regulates blood glucose to appropriate levels by promoting urinary excretion of glucose [2]. SGLT2 inhibitors act in an insulin-independent manner and are not associated with pancreatic β cell function. Therefore, SGLT2 inhibitors are attracting attention as alternative or combination therapies for type 2 diabetes. Since the approval of dapagliflozin by the European Medicines Agency as the first SGLT2 inhibitor in 2012, multiple other SGLT2 inhibitors have been developed, including canagliflozin, empagliflozin, ipragliflozin, tofogliflozin, luseogliflozin, and ertugliflozin [3,4,5]. These SGLT2 inhibitors are not only effective in glycemic control in type 2 diabetic patients but also effective in reducing cardiovascular events and improving renal outcomes [6,7,8,9,10]. The beneficial effect of empagliflozin, canagliflozin, and dapagliflozin treatment on the reduced hospitalization for heart failure and cardiovascular risk has been demonstrated in diabetic patients with heart failure from the multi-national clinical trials (i.e., the EMPA-REG OUTCOME trial, the CANVAS program, and the DECLARE-TIMI 58 trial) [6,7,8]. Dapagliflozin treatment has proved to reduce the risk of worsening heart failure in patients with heart failure regardless of the presence or absence of diabetes in DAPA-HF trials [11]. A similar clinical study with empagliflozin is under investigation in EMPA-TROPISM trial [12]. In patients with type 2 diabetes and kidney disease, the risk of kidney failure and cardiovascular event was lowered by the canagliflozin treatment [9,13] and by the empagliflozin treatment [10].

Improved glycemic control of SGLT2 inhibitors could be achieved by the decreased renal glucose resorption through the inhibition of SGLT2 [14]. Tahara et al. [3] reported that the urinary glucose excretion was increased by the treatment of six different SGLT2 inhibitors in a dose dependent manner but the duration time was varied. When compared the pharmacokinetic/pharmacodynamic features of these six SGLT2 inhibitors, the persistent duration of increased urinary glucose excretion and hypoglycemic control of dapagliflozin and ipragliflozin correlated with the kidney distribution of these SGLT2 inhibitors as well as their potent SGLT2 inhibition [3,6].

**Figure 1:**
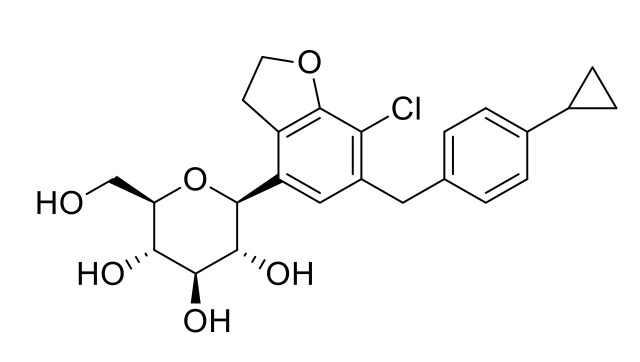
Chemical structure of DWP16001.

DWP16001 [(2S,3R,4R,5S,6R)-2-(7-chloro-6-(4-cyclopropylbenzyl)-2,3-dihydrobenzofuran-4-yl)- 6-(hydroxymethyl)tetrahydro-2*H*-pyran-3,4,5-triol] (Figure 1) is currently in a phase 2 clinical development program in Korea (Registration No. NCT04014023) as a new anti-diabetes drug with selective SGLT2 inhibitory effects. Choi et al., reported that DWP16001 is highly distributed in the kidney, which is the target organ; it showed selective and sustained SGLT2 inhibition (*IC*_50_, 0.8 ± 0.3 nM), compared to other drugs of its class, including dapagliflozin (*IC*_50_, 1.6 ± 0.3 nM) and ipragliflozin (*IC*_50_, 8.9 ± 1.7 nM) [15].

There is a need to characterize the comparative metabolism of a new drug and drug-metabolizing enzymes involved in metabolite formation for the prediction of its pharmacokinetics, potential drug–drug interactions, and toxicity [16,17,18]. In the present study, DWP16001 metabolism was compared among human, dog, mouse, monkey, and rat hepatocytes by liquid chromatography-high resolution mass spectrometry (LC-HRMS) and drug-metabolizing enzymes responsible for the metabolism of DWP16001 [i.e., cytochrome P450 (CYP) and UDP-glucuronosyltransferase (UGT)] were characterized using recombinant human CYP and UGT supersomes.

## 2. Materials and Methods

### 2.1. Chemicals and Reagents

DWP16001 (Batch No. E2016-085-27-2, purity 100.01%) and its authentic metabolite standards [i.e., (2S,3R,4R,5S,6R)-2-(7-chloro-6-(4-cyclopropylbenzyl)-2-hydroxy-2,3-dihydrobenzofuran-4-yl)-6-(hydroxymethyl)tetrahydro-2*H*-pyran-3,4,5-triol (M1); (2S,3R,4R,5S,6R)-2-(7-chloro-6-(4-(1- hydroxycyclopropyl)benzyl)-2,3-dihydrobenzofuran-4-yl)-6-(hydroxymethyl)tetrahydro-2*H*-pyran-3,4,5-triol (M2); 2-(3-chloro-4-(4-cyclopropylbenzyl)-2-hydroxy-6-((2S,3R,4R,5S,6R)-3,4,5-trihydroxy- 6-(hydroxymethyl)tetrahydro-2*H*-pyran-2-yl)phenyl)acetic acid (M3); and (2S,3S,4S,5R,6S)-6- (((2R,3S,4R,5R,6S)-6-(7-chloro-6-(4-cyclopropylbenzyl)-2,3-dihydrobenzofuran-4-yl)-3,4,5-trihydroxy-tetrahydro-2*H*-pyran-2-yl)methoxy)-3,4,5-trihydroxytetrahydro-2*H*-pyran-2-carboxylic acid (U3)] were given from Daewoong Pharmaceutical Co., Ltd. (Yongin, Korea). NADPH generating system was purchased from Promega (Madison, WI, USA). Uridine 5′-diphosphoglucuronic acid (UDPGA) were obtained from Sigma-Aldrich (St. Louis, MO, USA). LiverPool™ pooled human hepatocytes (50-donor mixed gender), INVITROGRO™ HT medium, and INVITROGRO™ KHB were from the products of BioIVT (Westbury, NY, USA). Mouse, rat, dog, and monkey cryopreserved hepatocytes; high viability cryopreserved recovery kit; ultrapooled human liver microsomes (150 donors); monkey liver microsomes; human recombinant UGT supersomes (1A1, 1A3, 1A4, 1A6, 1A7, 1A8, 1A9, 1A10, 2B4, 2B7, 2B10, 2B15, and 2B17); human recombinant CYP supersomes (1A1, 1A2, 2A6, 2B6, 2C8, 2C9, 2C19, 2D6, 2E1, 3A4, and 3A5); and anti-CYP3A4 antibody were the products of Corning Life Sciences (Woburn, MA, USA). Acetonitrile, methanol, and water (LC–MS grade) were obtained from Fisher Scientific Co. (Fair Lawn, NJ, USA). All other chemicals were of the highest quality available.

### 2.2. Metabolic Stability

To evaluate the metabolic stability of DWP16001 in human, dog, monkey, mouse, and rat hepatocytes, pooled cryopreserved hepatocytes were carefully thawed in recovery medium and resuspended in incubation medium (Krebs–Henseleit buffer for mouse, rat, and human; William’s E media for dog and monkey) to 0.5 × 10^6^ cells/mL for mouse, 0.67 × 10^6^ cells/mL for rat, and 1.0 × 10^6^ cells/mL for dog, monkey, and human in accordance with the manufacturer’s protocols. Then, 60-μL aliquots of these hepatocyte suspensions and an equal volume of 2 μM DWP16001 in incubation medium were mixed in 96-well plates and incubated in triplicate for 0, 30, 60, 90, 120, 180, or 240 min in a CO_2_ incubator at 37 °C. Reactions were stopped by addition of 120 µL of ice-cold acetonitrile to each well and the cell suspension was sonicated for 5 min at 4 °C, followed by centrifugation at 15,000× *g* for 10 min at 4 °C after 5 min of sonication. 80-µL aliquots of the supernatants were then vortex-mixed with 20 μL of ice-cold ketoconazole (an internal standard) in acetonitrile and analyzed by LC–tandem mass spectrometry (LC-MS/MS). The peak area ratios of DWP16001 versus internal standard at each sampling point were used for the subsequent calculation. Elimination parameters, including half-life (*t*_1/2_), intrinsic clearance (*Cl*_int_), and hepatic clearance (*Cl*_hep_) of DWP16001 in mouse, rat, dog, monkey, and human were calculated by the following equations [19].

(1) $$t_{1/2}\left(\min\right)=\frac{\ln2}{-k\;\left(\mathrm{elimination}\;\mathrm{slope}\right)}$$

(2) $$Cl_{\mathrm{int}}\left(\mathrm{mL}/\min/\mathrm{kg}\right)=\frac{\ln2}{t_{1/2}}\times \frac{\mathrm{mL}\;\mathrm{incubation}}{\mathrm{hepatocytes}\;\left(10^{6}\;\mathrm{cells}\right)}\times \frac{B\times 10^{6}\;\mathrm{cells}}{g\;\mathrm{liver}}\times \frac{A\;g\;\mathrm{liver}}{\mathrm{kg}\;\mathrm{BW}}$$

(3) $$Cl_{\mathrm{hep}}\left(\mathrm{mL}/\min/\mathrm{kg}\right)=\frac{Q_{h}\times Cl_{\mathrm{int}}}{Q_{h}+Cl_{\mathrm{int}}}$$

(4) $$\mathrm{Hepatic}\;\mathrm{extraction}\;\mathrm{ratio}\;=\;\frac{Cl_{\mathrm{hep}}}{Q_{h}}$$

Here, *A* has values of 87.5, 40, 32, 32, and 25.7; *B* has values of 135, 117, 215, 120, and 139; Q_h_ (representing hepatic blood flow) has values of 90, 55.2, 30.9, 43.4, and 20.7 mL/min/kg, for mouse, rat, dog, monkey, and human, respectively [20]. Hepatic extraction ratio values of ≤ 0.25, 0.25–0.75, and ≥ 0.75 were regarded as low, moderate, and high, respectively [19].

### 2.3. Metabolite Profiling in Human and Animal Hepatocytes

Sixty microliter aliquots of 10 μM DWP16001 in incubation medium and an equal volume of dog, monkey, mouse, rat, or human hepatocyte suspension were mixed in 96-well plates and incubated for 1 or 2 h in a CO_2_ incubator at 37 °C. Reactions were stopped by the addition of 120 µL of ice-cold acetonitrile to each well and the cell suspension was sonicated for 5 min at 4 °C, followed by centrifugation at 15,000× *g* for 10 min at 4 °C. The supernatants were then evaporated to dryness using a vacuum concentrator and residues were re-dissolved with 100 µL of 10% methanol. 5-μL aliquots of each sample were analyzed using LC–HRMS system.

### 2.4. Screening of CYP and UGT Enzymes Responsible for the Metabolism of DWP16001

DWP16001, M1, or M2 was incubated with 11 human cDNA-expressed CYP enzymes (1A1, 1A2, 2A6, 2B6, 2C8, 2C9, 2C19, 2D6, 2E1, 3A4, and 3A5) to identify the CYP enzymes involved in the metabolism of DWP16001. Reaction mixtures of 95 µL containing DWP16001 (final concentration: 5 µM), M1 or M2 (final concentration: 10 µM), and CYPs (final amount: 10 pmol) were prepared in 0.1 M potassium phosphate buffer (pH 7.4). The reaction was started by adding 5 µL of NADPH-generating system; the mixtures were incubated for 30 min at 37 °C in triplicate. Reactions were stopped by the addition of 400 µL of ice-cold ketoconazole (internal standard, 200 ng/mL) in ice-cold acetonitrile. Samples were centrifuged at 15,000× *g* for 8 min at 4 °C. Seventy-five microliter aliquots of the supernatants were diluted with 75 µL of deionized water and analyzed using an LC–MS/MS system.

To screen for UGT enzymes responsible for glucuronidation of DWP16001, 100 μL of reaction mixtures containing 13 human cDNA-expressed UGT enzymes (1A1, 1A3, 1A4, 1A6, 1A7, 1A8, 1A9, 1A10, 2B4, 2B7, 2B10, 2B15, and 2B17) at 0.1 mg protein/mL, 5 mM UDPGA, 0.025 mg/mL alamethicin, and 25 μM DWP16001 or M1 in 50 mM Tris buffer (pH 7.4) were incubated at 37 °C for 30 min in triplicate. Reactions were stopped by adding 100 μL of ketoconazole (internal standard, 200 ng/mL) in acetonitrile, followed by vortex-mixing and centrifugation (15,000× *g*, 8 min, 4 °C). The supernatant (75 μL) was diluted with equal volume of 30% acetonitrile and aliquots (5 μL) were injected into an LC-MS/MS system.

### 2.5. Immunoinhibition of DWP16001 Metabolism to M1 and M2 by anti-CYP3A4 Antibody

DWP16001 (5 μM) in human liver microsomes was incubated with varying concentrations of antibody specific for human CYP3A4 (anti-CYP3A4 antibody). Prior to incubation, human liver microsomes were pretreated with anti-CYP3A4 antibody for 15 min on ice. Reactions were initiated by addition of an aliquot of NADPH-generating system; mixtures were incubated for 60 min at 37 °C. Reactions were terminated by addition of 100 µL of ice-cold ketoconazole (200 ng/mL) in acetonitrile; following centrifugation at 15,000× *g* for 10 min at 4 °C. Aliquots of supernatant (150 µL) was evaporated using a vacuum concentrator and re-dissolved in 100 µL of 20% methanol; 5 µL aliquots were injected into an LC-MS/MS system.

### 2.6. Enzyme Kinetics for the Formation of M1 and M2 from DWP16001 in Human Liver Microsomes and cDNA-Expressed CYP3A4 or CYP2C19 Supersomes

To calculate the enzyme kinetic parameters for the M1 and M2 formation from DWP16001, DWP16001 (final concentrations, 1–200 μM) was incubated with NADPH-generating system, pooled human liver microsomes (0.25 mg/mL), or human cDNA-expressed CYP3A4 or CYP2C19 supersomes (10 pmol) at 37 °C for 30 min in triplicate. Reactions were stopped by addition of 400 μL of ice-cold ketoconazole (200 ng/mL) in acetonitrile. Following vortex-mixing and centrifugation (15,000× *g*, 8 min, 4 °C), 75 μL aliquots of the supernatant were diluted with 75 µL of 30% acetonitrile; 5 μL aliquots were injected into an LC−MS/MS system.

Apparent kinetic parameters (*K*_m_, *V*_max_ and *n*) for the formation of M1, M2, and M3 from DWP16001 by human liver microsomes, CYP3A4, or CYP2C19 were calculated as follows: the unweighted formation rates of M1, M2, and M3 over a range of DWP16001 concentrations were fitted to the Hill equation model [*V* = *V*_max_ × S^n^/(*K*_m_^n^ + S^n^)] or the single enzyme model [*V* = *V*_max_ × S/(*K*_m_ + S)] using Enzyme Kinetics software (version 1.1 SPSS Inc., Chicago, IL, USA). In the above equations, *V* means the reaction velocity of the formation of M1, M2, and M3 at given concentration [S] of DWP16001, *V*_max_ means the maximum reaction velocity, *n* means the Hill constant, and *K*_m_ means the DWP16001 concentration at which the reaction velocity is 50% of *V*_max_.

### 2.7. LC-MS Analysis

UFLC XR (Shimadzu, Kyoto, Japan) coupled with Q-Exactive Orbitrap (Thermo Scientific Inc., Waltham, MA, USA) was used for the identification of DWP16001 metabolites. Analytes were separated in Kinetex^®^ XB-C18 column (2.6 μm, 150 mm × 2.1 mm, Phenomenex, Torrance, CA, USA) using gradient elution of 0.1% formic acid in water (mobile phase A) and 0.1% formic acid in acetonitrile (mobile phase B). For profiling of phase I metabolites, gradient elution was performed as follows: 10% B for 0–0.2 min, 10–50% B for 0.2–47 min, 50% B held for 47–48 min, 50–10% B for 48–48.5 min, and 10% B for 48.5–60 min with the flow rate of 0.25 mL/min. For the profiling of phase II metabolites, gradient elution was performed as follows: 10% B for 0–2 min, 10–95% B for 2–11 min, 95% B held for 11–12 min, 95–10% B for 12–12.1 min, and 10% B for 12.1–15 min with the flow rate of 0.3 mL/min. MS spectra were acquired in positive-ion mode using electrospray ionization under optimized conditions as follows: capillary temperature, 350 °C; sheath gas flow rate, 60 (arbitrary units); aux gas flow rate, 25 (arbitrary units); and aux gas heater temperature, 100 °C. Full MS scan with data-dependent MS/MS was performed from *m*/*z* 100 to *m*/*z* 1000.

An Agilent 1290 Infinity LC coupled to a 6495 Triple Quadrupole MS (Agilent Technologies, Wilmington, DE, USA) was used for reaction phenotyping and metabolic stability analyses. Samples were separated in Halo^®^ C18 column (2.7 μm, 100 mm × 2.1 mm, Advanced Materials Technology, Wilmington, DE, USA) using gradient elution of mobile phase A and mobile phase B. The flow rate was 0.3 mL/min. For CYP reaction phenotyping, mobile phase was eluted in a gradient elution method: 10% B for 0–1.5 min, 10–60% B for 1.5–3 min, 60% B held for 3–4.5 min, 60–10% B for 4.5–4.6 min, and 10% B for 4.6–6 min. For UGT reaction phenotyping, mobile phase was eluted in a gradient elution method: 10% B for 0–1.5 min, 10–45% B for 1.5–3.5 min, 45% B held for 3.5–5.5 min, 45–10% B for 5.5–5.6 min, and 10% B for 5.6–7 min. Electrospray ionization conditions were optimized as follows: gas temperature, 200 °C; gas flow, 14 L/min; nebulizer, 40 psi; sheath gas temperature, 380 °C; sheath gas flow, 11 L/min; capillary voltage, 4500 V; and nozzle voltage, 500 V. Selective reaction monitoring transitions were 464.1→131.0 for DWP16001, 480.1→131.1 for M1, 480.1→209.0 for M2, 496.1→131.1 for M3, 640.1→131.0 for U1, 640.1→131.0 for U2, and 531.2→489.1 for ketoconazole (internal standard) at collision energies of 38, 40, 30, 34, 38, 40, and 34 eV, respectively.

## 3. Results

### 3.1. Metabolic Stability of DWP16001 in Hepatocytes

The in vitro metabolic stability parameters of DWP16001 using human, dog, monkey, mouse, and rat hepatocytes are shown in Table 1. The *t*_1/2_ values of DWP16001 in human, dog, monkey, mouse, and rat hepatocytes were 866.3, 223.5, 165.0, 533.1, and 990.0 min, respectively; *Cl*_int_ and *Cl*_hep_ values were 5.7 and 4.5 mL/min/kg, 42.7 and 17.9 mL/min/kg, 32.3 and 18.5 mL/min/kg, 61.4 and 36.5 mL/min/kg, and 9.8 and 8.3 mL/min/kg, respectively, using well-stirred model. The hepatic extraction ratios of DWP16001 in human, dog, monkey, mouse, and rat were 0.22, 0.56, 0.43, 0.41, and 0.15, respectively; these findings suggested that DWP16001 is weakly metabolized in the rat and human liver but is moderately metabolized in the dog, monkey, and mouse liver.

### 3.2. Metabolite Identification of DWP16001 in Hepatocytes

**Figure 2:**
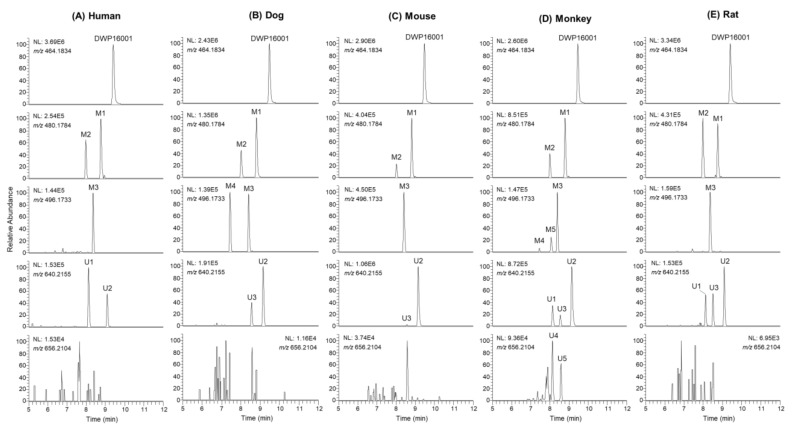
Extracted ion chromatograms of DWP16001 and its possible metabolites after incubation of 10 μM DWP16001 with (**A**) human, (**B**) dog, (**C**) mouse, (**D**) monkey, and (**E**) rat hepatocytes at 37 °C for 2 h.

DWP16001 was metabolized to five phase I metabolites (M1–M5) and 5 phase II metabolites (U1–U5) by incubation with human, dog, monkey, mouse, and rat hepatocytes. Figure 2 shows representative extracted ion chromatograms of DWP16001 and possible metabolites. The retention times, molecular formulae, observed molecular ions, mass errors, product ions, and biotransformation pathways of DWP16001 and its possible metabolites are summarized in Table 2.

**Figure 3:**
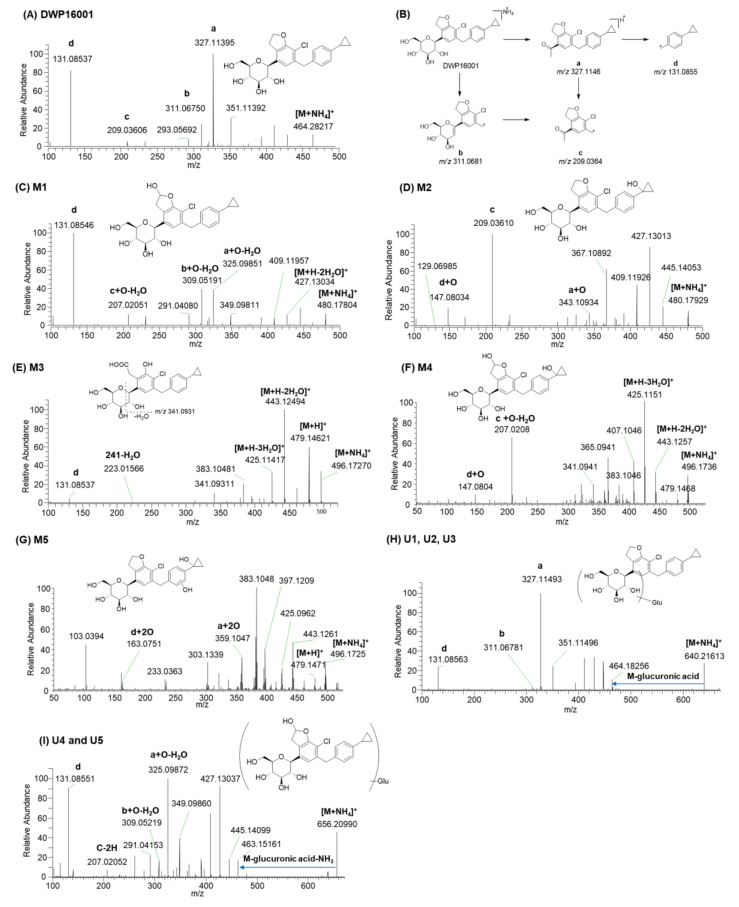
MS/MS spectra of (**A**) DWP16001 and (**C**–**I**) it’s possible metabolites and (**B**) the fragmentation mechanism of DWP16001.

DWP16001 showed an [M+NH_4_]^+^ ion at *m*/*z* 464.1829; the MS/MS spectrum of the *m*/*z* 464.1829 peak produced four characteristic product ions: *m*/*z* 327.1140 [a, 2-(4-bromo-2,5-dimethoxyphenyl)ethan-1-ylium], *m*/*z* 311.0675 (b, loss of cyclopropylbenzene moiety and H_2_O from [M+NH_4_]^+^), *m*/*z* 209.0361 (c, loss of cyclopropylbenzene moiety from *m*/*z* 327.1140), and *m*/*z* 131.0854 [d, (4-cyclopropylphenyl)methylium] (Figure 3A,B). Four major product ions served as markers of fragment ions for metabolite identification (Figure 3A).

M1 and M2 produced an [M+NH_4_]^+^ ion at *m*/*z* 480.1784, 15.9949 amu greater than the [M+NH_4_]^+^ ion of DWP16001, indicating that they were formed by monohydroxylation. M1 yielded product ions at *m*/*z* 427.1303 (loss of 2H_2_O and NH_3_ from [M+NH_4_]^+^), *m*/*z* 409.1195 (loss of H_2_O from *m*/*z* 427.1303), *m*/*z* 325.0985 (a + O − H_2_O), *m*/*z* 309.0519 (b + O − H_2_O), *m*/*z* 207.0205 (c + O − H_2_O), and *m*/*z* 131.0855 (d) (Figure 3C), suggesting that M1 was formed by monohydroxylation at the hydrofuran ring. M1 was identified as (2S,3R,4R,5S,6R)-2-(7-chloro-6-(4-cyclopropylbenzyl)-2-hydroxy-2,3- dihydrobenzofuran-4-yl)-6-(hydroxymethyl)tetrahydro-2*H*-pyran-3,4,5-triol (6-hydroxy-DWP16001) based on the retention time and MS/MS spectrum of the authentic standard.

M2 yielded product ions at *m*/*z* 445.1405 (loss of NH_3_ and H_2_O from [M+NH_4_]^+^), *m*/*z* 427.1301, *m*/*z* 409.1193, *m*/*z* 343.1093 (a + O), *m*/*z* 209.0361 (c), *m*/*z* 147.0803 (d + O), and *m*/*z* 129.0699 (loss of H_2_O from *m*/*z* 147.0803) (Figure 3D), indicating that M2 was formed by hydroxylation at the cyclopropyllbenzene moiety. M2 was identified as (2S,3R,4R,5S,6R)-2-(7-chloro-6-(4-(1- hydroxycyclopropyl)benzyl)-2,3-dihydrobenzofuran-4-yl)-6-(hydroxymethyl)tetrahydro-2*H*-pyran-3,4,5-triol, based on the retention time and MS/MS spectrum of the authentic standard.

M3 produced an [M+NH_4_]^+^ ion at *m*/*z* 496.1727, 31.9898 amu higher than that of DWP16001; it yielded product ions at *m*/*z* 479.1462 (loss of NH_3_ from [M+NH_4_]^+^), *m*/*z* 443.1249 (loss of 2H_2_O from *m*/*z* 479), *m*/*z* 425.1142 (loss of H_2_O from *m*/*z* 443), *m*/*z* 383.1048 (loss of CH_3_COOH from *m*/*z* 443), *m*/*z* 341.0931, and *m*/*z* 131.0855 (d) (Figure 3E). The presence of *m*/*z* 131 indicated that metabolism did not occur at the cyclopropyl benzene moiety. M3 was confirmed as 2-(3-chloro-4-(4-cyclopropylbenzyl)-2-hydroxy-6-((2S,3R,4R,5S,6R)-3,4,5-trihydroxy-6-(hydroxymethyl)tetrahydro-2*H*-pyran-2-yl) phenyl)acetic acid based on the retention time and MS/MS spectrum of the authentic standard. Incubation of M1 (10 μM) with human liver microsomes and NADPH resulted in the formation of M3, suggesting that M3 may be formed from DWP16001 via hydroxylation followed by oxidation of the furan ring.

M4 and M5 produced an [M+NH_4_]^+^ ion at *m*/*z* 496.1723, 31.9894 amu greater than the [M+NH_4_]^+^ of DWP16001, indicating dihydroxylation of DWP16001. M4 yielded product ions at *m*/*z* 479.1468, *m*/*z* 443.1257, *m*/*z* 425.1151, *m*/*z* 407.1046 (loss of H_2_O from *m*/*z* 425), *m*/*z* 207.0208 (c + O − H_2_O), and *m*/*z* 147.0804 (d + O) (Figure 3F), suggesting that M4 was formed by dihydroxylation at hydrofuran and cyclopropyl moieties. Incubation of M1 or M2 with monkey liver microsomes and NADPH at 37 °C resulted in the formation of M4 (data not shown). Based on these results, M4 was presumed to be (2S,3R,4R,5S,6R)-2-(7-chloro-2-hydroxy-6-(4-(1-hydroxycyclopropyl)benzyl)-2,3-dihydrobenzofuran -4-yl)-6-(hydroxymethyl)tetrahydro-2*H*-pyran-3,4,5-triol. M5 yielded product ions at *m*/*z* 479.1471, *m*/*z* 443.1261, *m*/*z* 425.0962, *m*/*z* 359.1047 (a + 2O), and *m*/*z* 163.0754 (d + 2O) (Figure 3G), indicating that M5 was produced by dihydroxylation at cyclopropylbenzene moiety. Incubation of M2 with monkey liver microsomes and NADPH at 37 °C resulted in the formation of M5 and M4. Based on these results, M5 was presumed to be hydroxy-M2, but an accurate position of hydroxylation at the benzene moiety of M5 could not be assigned.

U1–U3 produced an [M+NH_4_]^+^ ion at *m*/*z* 640.2155, 176.0321 amu greater than the [M+NH_4_]^+^ ion of DWP16001, indicating that they were DWP16001 glucuronides. U1, U2, and U3 yielded product ions at *m*/*z* 464.1826 (loss of glucuronic acid from [M+NH_4_]^+^), *m*/*z* 447.1581, *m*/*z* 327.1149 (a), *m*/*z* 311.0678 (b), and *m*/*z* 131.0855 (d) (Figure 3H). U3 was identified as glucuronidation at the 6-hydroxymethyl group in the tetrahydro-2*H*-pyran ring, based on the retention time and MS/MS spectrum of the authentic metabolite standard. U1 and U2 were identified as DWP16001 glucuronides but the accurate position of glucuronidation of each metabolite could not be determined. Glucuronidation of DWP16001 to U2 was detected in the hepatocytes of all five species; in contrast, U1 was detected in rat, monkey, and human hepatocytes alone, whereas U3 was detected in dog, monkey, mouse, and rat hepatocytes alone (Figure 2).

U4 and U5 produced an [M+NH_4_]^+^ ion at *m*/*z* 656.2099, 176.0321 amu greater than the [M+NH_4_]^+^ ion of hydroxy-DWP16001; they yielded product ions at *m*/*z* 463.1516 (loss of glucuronic acid and NH_3_ from M+NH_4_]^+^), *m*/*z* 445.1410 (loss of H_2_O from *m*/*z* 463), *m*/*z* 325.0987 (a + O − H_2_O), *m*/*z* 309.0522 (b + O − H_2_O), *m*/*z* 207.0205, and *m*/*z* 131.0855 (d) (Figure 3I). Incubation of M1 with monkey liver microsomes in the presence of UDPGA resulted in the formation of U4 and U5 (data not shown). Based on these results, U4 and U5 were identified as glucuronides of M1; however, the accurate position of glucuronidation of each metabolite was not characterized. U4 and U5 were detected only in monkey hepatocytes.

### 3.3. Characterization of Drug-Metabolizing Enzymes Responsible for DWP16001 Metabolism

**Figure 4:**
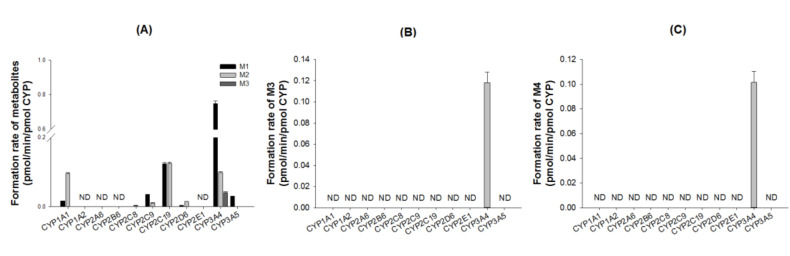
Formation rates of (**A**) M1, M2, and M3 from DWP16001 after incubation of 5 µM DWP16001, (**B**) M3 from M1 after incubation of 10 µM M1, and (**C**) M4 from M2 after incubation of 10 µM M2 with human recombinant CYP enzymes (1A1, 1A2, 2A6, 2B6, 2C8, 2C9, 2C19, 2D6, 2E1, 3A4, and 3A5) in the presence of NADPH. ND: not detected (lower limit of quantification: 0.003 pmol/min/pmol CYP).

To characterize the CYP enzymes responsible for the metabolism of DWP16001, the formation rates of M1, M2, and M3 from 5 µM DWP16001; M3 from 10 µM M1; M4 from 10 µM M2 were evaluated by incubation with 11 human recombinant CYP supersomes (1A1, 1A2, 2A6, 2B6, 2C8, 2C9, 2C19, 2D6, 2E1, 3A4, or 3A5) and NADPH. M1 was produced mainly by CYP3A4 with minor contributions of CYP2C19, CYP2C9, CYP3A5, CYP2D6, and CYP1A1 enzymes (Figure 4A). CYP2C19, CYP3A4, and CYP1A1 were important in the formation of M2 from DWP16001 (Figure 4A). M1 was metabolized to M3 by CYP3A4 (Figure 4B), but M4 was not detected after incubation of M1 with 11 human CYP enzymes and NADPH. The formation of M4 from M2 was catabolized by CYP3A4 alone (Figure 4C); M5 was not detected following incubation of M2 with 11 human CYP enzymes and NADPH.

**Figure 5:**
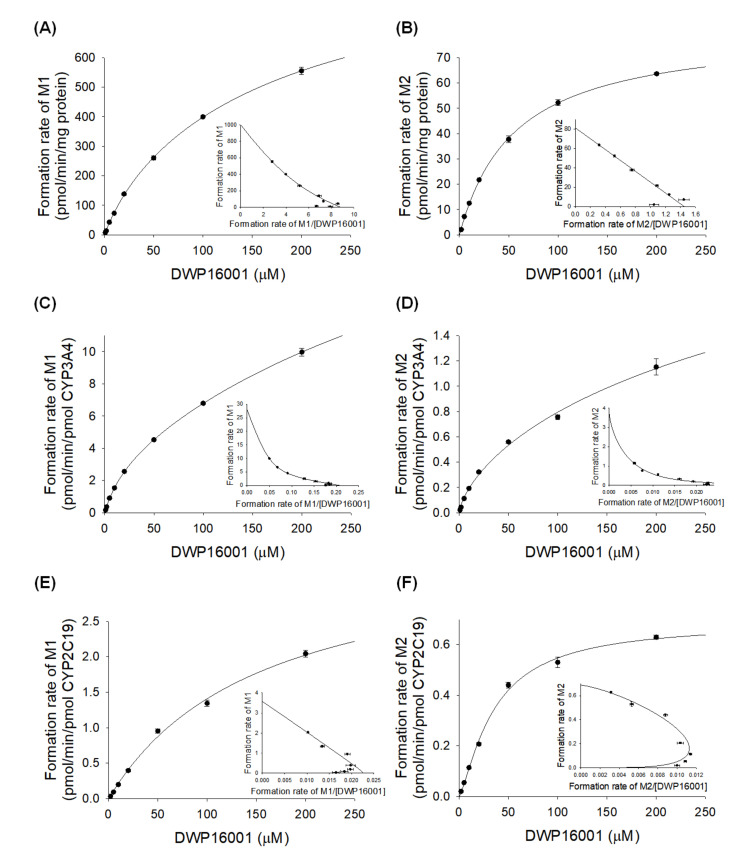
Michaelis–Menten plots for the formation of (**A**) M1 and (**B**) M2 from DWP16001 in human liver microsomes, (**C**) M1 and (**D**) M2 from DWP16001 in human cDNA-expressed CYP3A4 supersomes, and (**E**) M1 and (**F**) M2 from DWP16001 in human cDNA-expressed CYP2C19 supersomes. Insets are Eadie-Hofstee plots. Each data point represents the mean ± S.D. (*n* = 3).

The enzyme kinetic parameters for the M1 and M2 formation from DWP16001 in human liver microsomes and human recombinant CYP3A4 or CYP2C19 are summarized in Figure 5 and Table 3. The formation of M1 from DWP16001 in human liver microsomes and CYP3A4 fit the Hill equation with *K*_m_ values of 150.1 µM and 471.4 µM, respectively; it exhibited single enzyme kinetics with a *K*_m_ value of 156.4 µM in CYP2C19 supersomes (Table 3). The formation of M2 from DWP16001 in human liver microsomes, CYP3A4, and CYP2C19 fit the Hill equation with *K*_m_ values of 58.7 µM, 674.5 µM, and 35.4 µM, respectively (Table 3). The formation of M3 from DWP16001 in CYP3A4 supersomes exhibited single enzyme kinetics (*K*_m_, 10.4 µM; *V*_max_, 0.1278 μL/min/pmol CYP); M3 was not detected by enzyme kinetic analysis of DWP16001 in human liver microsomes and CYP2C19.

**Figure 6:**
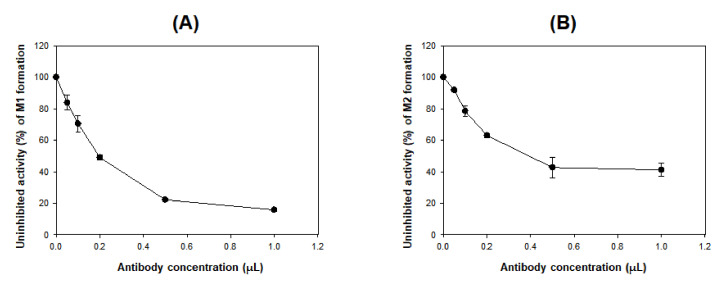
Effects of anti-CYP3A4 antibody on the formation of (**A**) M1 and (**B**) M2 from DWP16001 (5 μM) to in pooled human liver microsomes. Each data point represents the mean ± S.D. (*n* = 3).

As the concentration of CYP3A4 antibody increased in pooled human liver microsomes, the formation of M1 and M2 from DWP16001 decreased (Figure 6), supporting a major role for CYP3A4 in the formation of M1 and M2.

**Figure 7:**
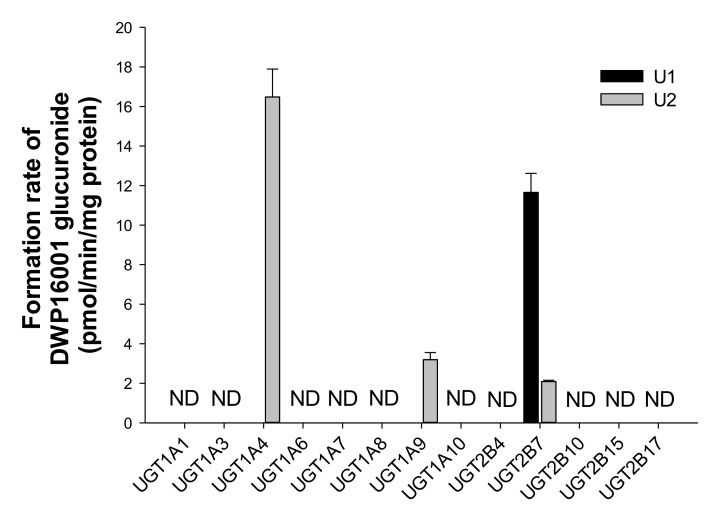
Formation rates of U1 and U2 from DWP16001 after incubation of 25 µM DWP16001 with human cDNA-expressed UGT1A1, UGT1A3, UGT1A5, UGT1A6, UGT1A7, UGT1A8, UGT1A9, UGT1A10, UGT2B4, UGT2B7, UGT2B10, UGT2B15, and UGT2B17 enzymes in the presence of UDPGA. Quantification was performed using the calibration curve of U3 because no authentic standards of U1 and U2 were available. ND: not detected (lower limit of quantification: 1.67 pmol/min/mg protein).

The glucuronidation of DWP16001 (25 µM) to U1 and U2 was evaluated by incubation with human cDNA-expressed UGT1A1, UGT1A3, UGT1A5, UGT1A6, UGT1A7, UGT1A8, UGT1A9, UGT1A10, UGT2B4, UGT2B7, UGT2B10, UGT2B15, and UGT2B17 in the presence of UDPGA. UGT2B7 played a prominent role in the glucuronidation of DWP16001 to U1, whereas UGT1A4, UGT1A9, and UGT2B7 were involved in the formation of U2 from DWP16001 (Figure 7). U3 was not formed by incubation of DWP16001 with 13 human UGT enzymes, supporting the lack of detection of U3 after incubation of DWP16001 with human hepatocytes (Figure 2).

## 4. Discussion

The metabolic stability of DWP16001 in human, dog, monkey, mouse, and rat hepatocytes resulted in hepatic extraction ratios of 0.22, 0.56, 0.43, 0.41, and 0.15, respectively; these findings indicated that DWP16001 is weakly metabolized in humans and rats, but moderately metabolized in dogs, monkeys, and mice (Table 1). The low hepatic extraction ratio in human and rat hepatocytes suggests that the hepatic clearance of DWP16001 is governed by the hepatic intrinsic clearance, in which DWP16001 metabolism is involved. On the other hand, moderate hepatic extraction ratio in dog, monkey, and mouse indicates that hepatic clearance of DWP16001 may depend on the intrinsic clearance and hepatic blood flow as well [21]. The comparison of metabolic stability of DWP16001 in hepatocytes from different species is very important for predicting human pharmacokinetics from those in experimental animals and for understanding the results of toxicity and pharmacokinetic experiments in different species. In terms of metabolic rate and hepatic clearance, it can be understood that rat shows the most similar patterns to humans than other experimental animals.

**Figure 8:**
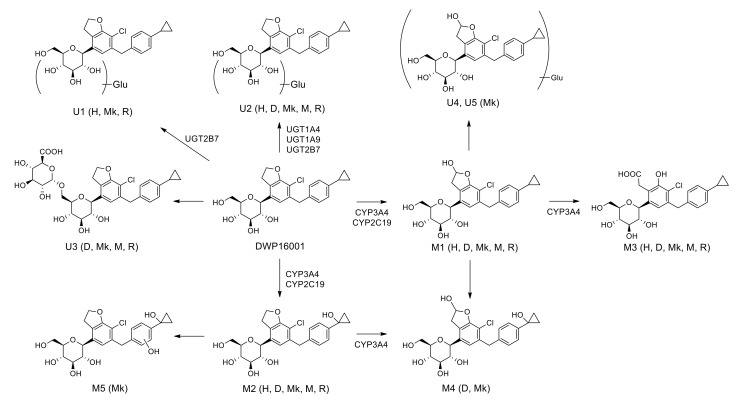
Possible in vitro metabolic pathways of DWP16001 in human, dog, monkey, mouse, and rat hepatocytes. H: human; D: dog; Mk: monkey; M: mouse; R: rat; Glu: glucuronide.

Five phase I metabolites (M1–M5), three DWP16001 glucuronides (U1–U3), and two hydroxy-DWP16001 (M1) glucuronides (U4 and U5) were identified in five species hepatocytes were shown in Figure 8. Based on investigation of the metabolizing enzymes responsible for the formation of phase I and glucuronide metabolites, as well as subsequent metabolism from M1 to M3 and from M2 to M4 and M5 using human and monkey liver microsomes, the proposed in vitro metabolic pathways of DWP16001 and responsible metabolic enzymes are shown in Figure 8.

DWP16001 was metabolized to M1 by hydroxylation at the furan moiety, M2 by hydroxylation at the cyclopropyl moiety, and M3 by oxidation of M1 at hydrofuran moiety in human, dog, monkey, mouse, and rat hepatocytes; M1–M3 were identified based on the authentic standard. Incubation of M1 and M2 with monkey liver microsomes and NADPH resulted in the formation of M4, which was detected in dog and monkey hepatocytes. M5 was only detected in monkey hepatocytes; it was formed by hydroxylation at the benzene moiety after incubation of M2 with monkey liver microsomes. Among three DWP16001 glucuronides (U1–U3), U3 was formed by glucuronidation of DWP16001 at the 6-hydroxymethyl group of the tetrahydro-2*H*-pyran ring; however, accurate glucuronidation positions of U1 and U2 could not be identified because of the lack of authentic standards. U2 was detected in hepatocytes from all species; conversely, U1 was detected in human, monkey, and rat hepatocytes alone, whereas U3 was identified in dog, monkey, mouse, and rat hepatocytes alone (Figure 2). Incubation of M1 with monkey liver microsomes in the presence of UDPGA resulted in the formation of U4 and U5, which were detected only in monkey hepatocytes. DWP16001 in human and rat hepatocytes showed limited metabolism and similar metabolite profiles relative to dog, monkey, and mouse hepatocytes. Glucuronidation of DWP16001 at the tetrahydropyran moiety was similar to glucuronidation of other SGLT2 inhibitors such as canagliflozin, dapagliflozin, ertugliflozin, empagliflozin, and luseogliflozin [22,23,24,25,26].

The intrinsic clearance (*Cl*_int_, *V*_max_/*K*_m_) for the formation of M1 and M2 from DWP16001 were 6.531 and 1.411 μL/min/mg protein, respectively, in human liver microsomes, 0.061 and 0.006 μL/min/pmol CYP, respectively, in CYP3A4 supersomes, and 0.023 and 0.019 μL/min/pmol CYP, respectively, in CYP2C19 supersomes (Table 3). *Cl*_int_ value for the formation of M3 from DWP16001 in CYP3A4 supersomes was 0.012 μL/min/pmol CYP. These results indicated that hydroxylation of DWP16001 to M1 was more favorable metabolic pathway than hydroxylation to M2 and M3. CYP3A4 and CYP2C19 were responsible for the metabolism of DWP16001 to M1 and M2. Immunoinhibition study using anti-CYP3A4 antibody supported the major role of CYP3A4 in the formation of M1 and M2 from DWP16001 (Figure 6). The metabolism of M1 to M3 and metabolism of M2 to M4 were catalyzed only by CYP3A4 (Figure 4B,C). The formation of M4 from M1 and M5 from M2 was not observed after incubation of M1 or M2 with 11 human CYP enzymes. These results confirmed that M4 and M5 were not detected as metabolites of DWP16001 in human hepatocytes.

Among 13 human UGT enzymes examined, the glucuronidation of DWP16001 to U1 was catalyzed by UGT2B7, whereas the formation of U2 from DWP16001 was catalyzed by UGT1A4, UGT1A9, and UGT2B7 (Figure 7). However, DWP16001 glucuronide, U3 and two M1 glucuronides, U4 and U5 were not formed by incubation of DWP16001 or M1 with 13 human UGT enzymes and UDPGA. These results supported identification of U1 and U2; U3, U4, and U5 were not detected following incubation of DWP16001 with human hepatocytes.

The metabolic pattern of DWP16001 is similar to that of previously known SGLT2 inhibitors. Dapagliflozin showed low hepatic extraction in human hepatocytes and Major phase I metabolites were catalyzed by multiple CYP enzymes with the highest contribution of CYP2D6, CYP3A4, and CYP2C9 [27,28]. UGT1A9 was involved in the formation of dapagliflozin-3-*O*-glucuronide, a major in vivo metabolite of dapagliflozin [23,28]. Canagliflozin was extensively metabolized by UGT1A9 and UGT2B4-catalyzed *O*-glucuronidation and via CYP3A4-catalyzed hydroxylation [22,29]. Similarly, ertugliflozin was extensively metabolized via *O*-glucuronidation by UGT1A9 and UGT2B7 and via hydroxylation by CYP3A4/3A5 and CYP2D6 [24,30]. Bexagliflozin undergoes oxidation and glucuronidation to form six principal metabolites in humans, and the metabolism is primarily mediated by CYP3A4 and UGT1A9 [31].

The involvement of CYP and UGT enzymes could cause drug–drug interaction with SGLT2 inhibitors. In the case of dapagliflozin, dapagliflozin-3-*O*-glucuronide is a major in vivo metabolite of dapagliflozin which was mediated by UGT1A9 but oxidative metabolism of dapagliflozin catalyzed by CYP2D6, CYP3A4, and CYP2C9 etc. accounted for less than 10% of dapagliflozin metabolism in human [27,28]. Therefore, repeated rifampin administration (600 mg/day for 6 days), which was used as an inducer of CYP and UGT enzymes, decreased area under plasma concentration curve (*AUC*) of dapagliflozin by 22% as a result of the induction of UGT1A9 in human [32]. Pretreatment of mefenamic acid (250 mg every 6 h for 5 days), a strong UGT1A9 inhibitor, increased the *AUC* of dapagliflozin by 50% in human [32]. However, co-administration of pioglitazone, glimepiride, and simvastatin, which are frequently prescribed with anti-diabetic and cardiovascular drugs and are metabolized by CYP2C8, CYP2C9, and CYP3A4, respectively, did not cause significant drug interactions with dapagliflozin [33,34]. Similarly, repeated rifampin administration (600 mg/day for 8 days) decreased *AUC* of canagliflozin by 48.7% and co-administration of probenecid (500 mg, twice per day for 3 days), an inhibitor of UGT enzymes, increased canagliflozin *AUC* by 20.7% in human [35,36]. Co-administration of cyclosporine A (400 mg), an inhibitor of p-glycoprotein, CYP3A4, and OATP1B1, increased canagliflozin *AUC* by 23% in human [35,36]. However, glyburide, metformin, and simvastatin that are frequently prescribed with anti-diabetic and cardiovascular drugs, did not cause significant drug interactions with canagliflozin [36,37]. These results suggest that, in case of DWP16001, the inhibitors of CYP3A4 (e.g., clarithromycin, cobicistat, erythromycin, ritonavir, indinavir, and itraconazole), CYP2C19 (e.g., fluvoxamine, fluconazole, and ticlopidine), UGT1A4, UGT1A9 (e.g., mefenamic acid), and UGT2B7 (e.g., valproic acid) or inducer of CYP and UGT enzymes (e.g., rifampin) could cause the pharmacokinetic drug–drug interactions with DWP16001 [38]. Therefore, further investigation on the effect of these inhibitors or inducers on the metabolism and pharmacokinetics of DWP16001 is necessary, followed by the elucidation of the contribution of these CYP3A4, CYP2C19, UGT1A4, UGT1A9, and UGT2B7 to the in vivo metabolism and pharmacokinetics of DWP16001.

In conclusion, DWP16001 was metabolized to five phase 1 metabolites (M1–M5) by hydroxylation and alcohol oxidation, three DWP16001 glucuronides (U1–U3), and two M1 glucuronides (U4–U5) in human, dog, monkey, mouse, and rat hepatocytes. Incubation of DWP16001 with pooled human hepatocytes showed limited metabolism and resulted in the formation of M1, M2, M3, U1, and U2. CYP3A4, CYP2C19, UGT1A4, UGT1A9, and UGT2B7 were responsible for the metabolism of DWP16001. Although DWP16001 is weakly metabolized in human hepatocytes, there is a potential for modification of pharmacokinetics and drug–drug interactions, considering the enzymes involved in its metabolism.

## Figures and Tables

**Table 1:** Half-life (*t*_1/2_), intrinsic clearance (*Cl*_int_), hepatic clearance (*Cl*_hep_), and hepatic extraction ratio values of DWP16001 in human, dog, monkey, mouse, and rat hepatocytes.

Parameters	Human	Dog	Monkey	Mouse	Rat
*t*_1/2_ (min)	866.3	223.5	165.0	533.1	990.0
*Cl*_int_ (mL/min/kg)	5.7	42.7	32.3	61.4	9.8
*Cl*_hep_ (mL/min/kg)	4.5	17.9	18.5	36.5	8.3
Hepatic extraction ratio	0.22	0.56	0.43	0.41	0.15

**Table 2:** Retention time (t_R_), molecular formulae, observed molecular ions, mass errors, product ions, and biotransformation of DWP16001 and its metabolites identified in mouse, rat, dog, monkey, and human hepatocytes.

Compound	t_R_ (min)	Elemental Composition	Observed [M+NH_4_]^+^ (*m*/*z*)	Mass Error (ppm)	Product Ions (*m*/*z*)	Biotransformation
DWP16001	9.4	C_24_H_31_O_6_NCl	464.1829	−1.08	327.1140, 311.0675, 209.0361, 131.0854	
M1	8.8	C_24_H_31_O_7_NCl	480.1781	−0.62	427.1303, 409.1196, 325.0985, 309.0519, 207.0205, 131.0855	Monohydroxylation
M2	8.0	C_24_H_31_O_7_NCl	480.1793	1.87	445.1405, 427.1301, 409.1193, 343.1093, 209.0361, 147.0803	Monohydroxylation
M3	8.4	C_24_H_31_O_8_NCl	496.1727	−1.21	479.1462, 443.1249, 425.1142, 383.1048, 341.0931, 131.0854	Hydroxylation + oxidation
M4	7.4	C_24_H_31_O_8_NCl	496.1723	−2.02	479.1468, 443.1257, 425.1151, 407.1046, 207.0208, 147.0804	Dihydroxylation
M5	8.0	C_24_H_31_O_8_NCl	496.1728	-1.01	479.1471, 443.1261, 425.0962, 359.1047, 163.0751	Dihydroxylation
U1	8.1	C_30_H_39_O_12_NCl	640.2161	0.94	464.1826, 327.1149, 311.0678, 131.0856	Glucuronidation
U2	9.1	C_30_H_39_O_12_NCl	640.2145	−1.56	447.1571, 327.1149, 311.0680, 131.0857	Glucuronidation
U3	8.6	C_30_H_39_O_12_NCl	640.2150	−0.78	464.1833, 447.1566, 327.1143, 311.0678, 131.0854	Glucuronidation
U4	8.1	C_30_H_39_O_13_NCl	656.2099	−0.76	463.1516, 445.1410, 325.0987, 309.0522, 207.0205, 131.0855	Hydroxylation + glucuronidation
U5	8.6	C_30_H_39_O_13_NCl	656.2099	−0.76	445.1397, 325.0987, 309.0522, 207.0206, 131.0855	Hydroxylation + glucuronidation

**Table 3:** Enzyme kinetic parameters for the metabolism of DWP16001 to M1 and M2 in human liver microsomes (HLM) and human recombinant CYP3A4 and CYP2C19 supersomes.

Enzymes	M1 Formation					M2 Formation				
	*K*_m_ (µM)	*V*_max_	*Cl*_int_	*n*	Mode	*K*_m_ (µM)	*V*_max_	*Cl*_int_	*n*	Mode
HLM	150.1	980.3	6.531	0.9176	Hill	58.7	82.8	1.411	0.9773	Hill
CYP3A4	471.4	28.9	0.061	0.7505	Hill	674.5	3.8	0.006	0.696	Hill
CYP2C19	156.4	3.6	0.023	-	Single enzyme	35.4	0.688	0.019	1.3	Hill

*V*_max_: HLM, pmol/min/mg protein; CYP3A4 and CYP2C19, pmol/min/pmol CYP; *Cl_i_*_nt_ (*V*_max_/*K*_m_): HLM, μL/min/mg protein; CYP3A4 and CYP2C19, μL/min/pmol CYP; *n*: hill coefficient.

## References

[1] International Diabetes Federation (2019). IDF Diabetes Atlas.

[2] Tat V., Forest C.P. (2018). The role of SGLT2 inhibitors in managing type 2 diabetes. JAAPA.

[3] Tahara A., Takasu T., Yokono M., Imamura M., Kurosaki E. (2016). Characterization and comparison of sodium-glucose cotransporter 2 inhibitors: Part 2. Antidiabetic effects in type 2 diabetic mice. J. Pharmacol. Sci..

[4] Ito H., Shinozaki M., Nishio S., Abe M. (2016). SGLT2 inhibitors in the pipeline for the treatment of diabetes mellitus in Japan. Expert Opin. Pharmacother..

[5] Nauck M.A. (2014). Update on developments with SGLT2 inhibitors in the management of type 2 diabetes. Drug Des. Dev. Ther..

[6] Neal B., Perkovic V., Mahaffey K.W., de Zeeuw D., Fulcher G., Erondu N., Shaw W., Law G., Desai M., Matthews D. (2017). Canagliflozin and cardiovascular and renal events in type 2 diabetes. N. Engl. J. Med..

[7] Zinman B., Wanner C., Lachin J.M., Fitchett D., Bluhmki E., Hantel S., Mattheus M., Devins T., Johansen O.E., Woerle H.J. (2015). Empagliflozin, cardiovascular outcomes, and mortality in type 2 diabetes. N. Engl. J. Med..

[8] Wiviott S.D., Raz I., Bonaca M.P., Mosenzon O., Kato E.T., Cahn A., Silverman M.G., Zelniker T.A., Kuder J.F., Murphy S.A. (2018). Dapagliflozin and cardiovascular outcomes in type 2 diabetes. N. Engl. J. Med..

[9] Perkovic V., de Zeeuw D., Mahaffey K.W., Fulcher G., Erondu N., Shaw W., Barrett T.D., Weidner-Wells M., Deng H., Matthews D.R. (2018). Canagliflozin and renal outcomes in type 2 diabetes: Results from the CANVAS Program randomised clinical trials. Lancet Diabetes Endocrinol..

[10] Wanner C., Inzucchi S.E., Lachin J.M., Fitchett D., von Eynatten M., Mattheus M., Johansen O.E., Woerle H.J., Broedl U.C., Zinman B. (2016). Empagliflozin and progression of kidney disease in type 2 diabetes. N. Engl. J. Med..

[11] McMurray J.J.V., Solomon S.D., Inzucchi S.E., Køber L., Kosiborod M.N., Martinez F.A., Ponikowski P., Sabatine M.S., Anand I.S., Bělohlávek J. (2019). Dapagliflozin in patients with heart failure and reduced ejection fraction. N. Engl. J. Med..

[12] Santos-Gallego C.G., Garcia-Ropero A., Mancini D., Pinney S.P., Contreras J.P., Fergus I., Abascal V., Moreno P., Atallah-Lajam F., Tamler R. (2019). Rationale and design of the EMPA-TROPISM trial (ATRU-4): Are the “Cardiac Benefits” of empagliflozin independent of its hypoglycemic activity?. Cardiovasc. Drugs Ther..

[13] Perkovic V., Jardine M.J., Neal B., Bompoint S., Heerspink H.J.L., Charytan D.M., Edwards R., Agarwal R., Bakris G., Bull S. (2019). Canagliflozin and Renal Outcomes in Type 2 Diabetes and Nephropathy. N. Engl. J. Med..

[14] Garcia-Ropero A., Badimon J.J., Santos-Gallego C.G. (2018). The pharmacokinetics and pharmacodynamics of SGLT2 inhibitors for type 2 diabetes mellitus: The latest developments. Expert Opin. Drug Metab. Toxicol..

[15] Choi M.-K., Nam S.J., Ji H.-Y., Park M.J., Choi J.-S., Song I.-S. (2020). Comparative pharmacokinetics and pharmacodynamics of a novel sodium-glucose cotransporter 2 inhibitor, DWP16001, with dapagliflozin and ipragliflozin. Pharmaceutics.

[16] Foti R.S., Dalvie D.K. (2016). Cytochrome P450 and non-cytochrome P450 oxidative metabolism: Contributions to the pharmacokinetics, safety and efficacy of xenobiotics. Drug Metab. Dispos..

[17] Cerny M.A. (2016). Prevalence of non-cytochrome P450-mediated metabolism in Food and Drug Administration-approved oral and intravenous drugs: 2006–2015. Drug Metab. Dispos..

[18] Hwang D., Kim J.-H., Shin Y., Choi W.G., Kim S., Ho Y.-Y., Lee J.Y., Kang H.C., Lee H.S. (2019). Identification of catalposide metabolites in human liver and intestinal preparations and characterization of the relevant sulfotransferase, UDP-glucuronosyltransferase, and carboxylesterase enzymes. Pharmaceutics.

[19] Bohnert T., Gan L.S., Chuang Lu A.P.L. (2010). The role of drug metabolism in drug discovery. Enzyme Inhibition in Drug Discovery and Development: The Good and the Bad.

[20] Davies B., Morris T. (1993). Physiological parameters in laboratory animals and humans. Pharm. Res..

[21] Atkinson A.J., Kushner W. (1979). Clinical pharmacokinetics. Ann. Rev. Pharmacol. Toxicol..

[22] Mamidi R.N.V.S., Cuyckens F., Chen J., Scheers E., Kalamaridis D., Lin R., Silva J., Sha S., Evans D.C., Kelley M.F. (2014). Metabolism and excretion of canagliflozin in mice, rats, dogs, and humans. Drug Metab. Dispos..

[23] Obermeier M., Yao M., Khanna A., Koplowitz B., Zhu M., Li W., Komoroski B., Kasichayanula S., Discenza L., Washburn W. (2010). In vitro characterization and pharmacokinetics of dapagliflozin (BMS-512148), a potent sodium-glucose cotransporter Type II inhibitor, in animals and humans. Drug Metab. Dispos..

[24] Miao Z., Nucci G., Amin N., Sharma R., Mascitti V., Tugnait M., Vaz A.D., Callegari E., Kalgutkar A.S. (2013). Pharmacokinetics, metabolism, and excretion of the antidiabetic agent ertugliflozin (PF-04971729) in healthy male subjects. Drug Metab. Dispos..

[25] Miyata A., Hasegawa M., Hachiuma K., Mori H., Horiuchi N., Mizuno-Yasuhira A., Chino Y., Jingu S., Sakai S., Samukawa Y. (2017). Metabolite profiling and enzyme reaction phenotyping of luseogliflozin, a sodium–glucose cotransporter 2 inhibitor, in humans. Xenobiotica.

[26] Chen L.-Z., Jungnik A., Mao Y., Philip E., Sharp D., Unseld A., Seman L., Woerle H.-J., Macha S. (2015). Biotransformation and mass balance of the SGLT2 inhibitor empagliflozin in healthy volunteers. Xenobiotica.

[27] Kasichayanula S., Liu X., LaCreta F., Griffen S.C., Boulton D.W. (2014). Clinical Pharmacokinetics and Pharmacodynamics of Dapagliflozin, a selective inhibitor of sodium-glucose co-transporter Type 2. Clin. Pharmacokinet..

[28] Meng W., Ellsworth B.A., Nirschl A.A., McCann P.J., Patel M., Girotra R.N., Wu G., Sher P.M., Morrison E.P., Biller S.A. (2008). Discovery of dapagliflozin: A potent, selective renal sodium-dependent glucose cotransporter 2 (SGLT2) inhibitor for the treatment of type 2 diabetes. J. Med. Chem..

[29] Dong S.T., Niu H.M., Wu Y., Jiang J.L., Li Y., Jiang K.Y., Wang X., Zhang M.F., Han M.F., Meng S.N. (2018). Plasma pharmacokinetic determination of canagliflozin and its metabolites in a type 2 diabetic rat model by UPLC-MS/MS. Molecules.

[30] Kalgutkar A.S., Tugnait M., Zhu T., Kimoto E., Miao Z., Mascitti V., Yang X., Tan B., Walsky R.L., Chupka J. (2011). Preclinical species and human disposition of PF-04971729, a selective inhibitor of the sodium-dependent glucose cotransporter 2 and clinical candidate for the treatment of type 2 diabetes mellitus. Drug Metab. Dispos..

[31] Zhang W., Li X., Ding H., Lu Y., Stilwell G.E., Halvorsen Y.D., Welihinda A. (2020). Metabolism and disposition of the SGLT2 inhibitor bexagliflozin in rats, monkeys and humans. Xenobiotica.

[32] Kasichayanula S., Liu X., Griffen S.C., Lacreta F.P., Boulton D.W. (2013). Effects of rifampin and mefenamic acid on the pharmacokinetics and pharmacodynamics of dapagliflozin. Diabetes Obes Metab..

[33] Kasichayanula S., Liu X., Shyu W.C., Zhang W., Pfister M., Griffen S.C., Li T., LaCreta F.P., Boulton D.W. (2011). Lack of pharmacokinetic interaction between dapagliflozin, a novel sodium-glucose transporter 2 inhibitor, and metformin, pioglitazone, glimepiride or sitagliptin in healthy subjects. Diabetes Obes. Metab..

[34] Kasichayanula S., Chang M., Liu X., Shyu W.C., Griffen S.C., LaCreta F.P., Boulton D.W. (2012). Lack of pharmacokinetic interactions between dapagliflozin and simvastatin, valsartan, warfarin, or digoxin. Adv. Ther..

[35] Devineni D., Vaccaro N., Murphy J., Curtin C., MamidiRao N., Weiner S., Wang S.S., Ariyawansa J., Stieltjes H., Wajs E. (2015). Effects of rifampin, cyclosporine A, or probenecid on the pharmacokinetic profile of canagliflozin, a sodium glucose co-transporter 2 inhibitor, in healthy participants. Int. J. Clin. Pharmacol. Ther..

[36] Devineni D., Polidori D. (2015). Clinical pharmacokinetic, pharmacodynamic, and drug–drug interaction profile of canagliflozin, a sodium-glucose co-transporter 2 inhibitor. Clin. Pharmacokinet..

[37] Devineni D., Manitpisitkul P., Murphy J., Skee D., Wajs E., Mamidi R.N., Tian H., Vandebosch A., Wang S.S., Verhaeghe T. (2015). Effect of canagliflozin on the pharmacokinetics of glyburide, metformin and simvastatin in healthy participants. Clin. Pharm. Drug Dev..

[38] Tornio A., Filppula A.M., Niemi M., Backman J.T. (2019). Clinical studies on drug-drug interactions involving metabolism and transport: Methodology, pitfalls, and interpretation. Clin. Pharmacol. Ther..

